# “True” Lateral Imaging for Cervical Medial Branch Radiofrequency Neurotomy

**DOI:** 10.1093/pm/pnaa391

**Published:** 2020-11-08

**Authors:** Patrick H Waring

**Affiliations:** Pain Intervention Center, Metairie, Louisiana, USA

The effective use of radiofrequency thermal energy for the palliative relief of cervical facet pain has been known for decades [1]. The radiofrequency cannula is ideally placed in an exact manner both parallel to and as close as possible to the targeted cervical medial branch nerve before lesioning [2]. “True” lateral cervical imaging has previously been described and is characterized by four criteria: location of bilateral transverse processes in the posterior/superior quadrant of the vertebral body, maintenance of clear disk spaces, creation of maximal distance between the posterior edge of the articular pillar and the base of the spinous process, and superimposition of bilateral articular pillars [3]. Such imaging allows accurate cannula tip placement in the articular pillar lesion zone [3].

As illustrated by the following images obtained from a single cadaveric specimen, a simple process yields precise “true” lateral imaging during cannula positioning [4]. First, the subject’s head is placed in a neutral, nonrotated position (Figure 1A). Second, the lateral fluoroscope is positioned such that the image intensifier is estimated to be perpendicular to both the longitudinal and axial cervical planes (Figure 1B). Third, after initial imaging, the fluoroscope is rotated axially so that the bilateral articular pillar posterior aspects are superimposed (Figure 2A–2C). The fluoroscope is then rotated longitudinally so that the bilateral superior/inferior aspects are superimposed (Figure 2D–2F). Through the use of lateral fluoroscopic imaging, the cannula is then advanced anteriorly along the lateral aspect of the articular pillar until the active tip is parallel and close to the targeted nerve. In summary, the above technique produces clear “true” lateral cervical imaging during radiofrequency neurotomy.

**Figure 1:**
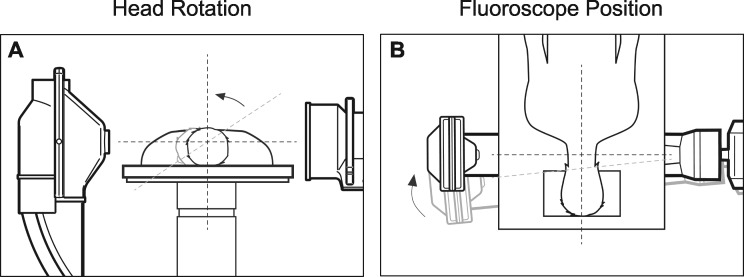
Head and fluoroscope positions. (**A**) Head rotation. Patient is in prone position. Head in non-neutral position (outlined in gray) prevents true lateral imaging. After head rotation (head outlined in black and rotation indicated by arrow), neutral position facilitates true lateral imaging. (**B**) Fluoroscope position. Fluoroscope (outlined in gray) not perpendicular to neck adversely affects imaging. After fluoroscope (outlined in black) is positioned perpendicular to neck, lateral imaging is facilitated.

**Figure 2.**
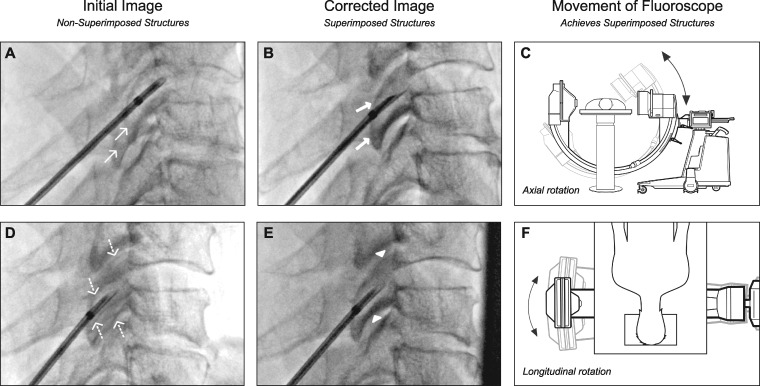
Axial/longitudinal fluoroscopic rotation during C6 medial branch neurotomy. (**A**) Thin arrows indicate non-superimposed posterior pillar aspects. (**B**) Thick arrows indicate superimposed posterior pillar aspects after (**C**) axial rotation. (**D**) Dashed arrows indicate non-superimposed superior/inferior pillar aspects. (**E**) Arrowheads indicate superimposed superior/inferior pillar aspects after (**F**) longitudinal rotation. All radiographic images were obtained from a cadaveric specimen donated by the Spine Intervention Society.

## Notes

Funding sources: The Spine Intervention Society donated the cadaveric specimen.
